# Genomic study of adolescent idiopathic scoliosis in Japan

**DOI:** 10.1186/s13013-016-0067-x

**Published:** 2016-04-01

**Authors:** Shiro Ikegawa

**Affiliations:** Laboratory for Bone and Joint Diseases, RIKEN Center for Integrative Medical Sciences, 4-6-1 Shirokanedai, Minato-ku, Tokyo, 108-8639 Japan

**Keywords:** Genetics, Adolescent idiopathic scoliosis, GWAS, Japan, *LBX1*, *GPR126*, *BNC2*

## Abstract

Adolescent idiopathic scoliosis (AIS) is a common disease. It is a multi-factorial (polygenic) disease controlled by genetic and environmental factors. Studies searching for genetic factors of AIS using linkage and association analyses have been conducted and several susceptibility genes have been reported. This paper reviews the recent progress in the genome-wide association study of AIS in Japan and comments on its future task.

## Background

Scoliosis is a common spinal deformity. It is a lateral bending of the spine diagnosed by using the antero-posterior plane radiograph of the whole spine in the standing position; however the deformity (spinal curve) is actually 3-dimentional. The degree of spinal curve is evaluated by the Cobb angle. The Cobb angle of more than 10–15° is considered pathological (disease) since some degree of scoliosis is commonly seen in the general population.

Scoliosis is a mysterious disease. It can be congenital. A variety of spinal deformities including hemi-vertebra and block vertebra results in scoliosis. Scoliosis can be neuromuscular. A lot of conditions could lead to scoliosis, including cerebral palsy, spina bifida, polio, and muscular dystrophy. Scoliosis can also be syndrome-related. Connective tissue diseases like Marfan syndrome and Ehlers-Danlos syndrome, skeletal dysplasias, and neurofibromatosis present with scoliosis. However, the most common type of scoliosis is idiopathic. Specific cause is unknown in over 80 % of all scoliosis cases [1].

Idiopathic scoliosis is classified into three types, *i.e.,* infantile, juvenile and adolescent scoliosis. Adolescent idiopathic scoliosis (AIS) is the most common and the most mysterious form of scoliosis. If the Cobb angle of ≧10° is a criterion, ~1 % of children are suffering from it in Japan [1]. Previously normal children suddenly come to suffer from progressive bending of the spine during their adolescence. There is also a strong female predominance.

### GWAS

To solve the mystery of scoliosis, of AIS in particular, a group of expert scoliosis surgeons in Japan stood up and made a consortium, the Japan Scoliosis Clinical Research Group (JSCRG). This consortium was launched in January 31, 2009, supported by a research grant from Japanese Orthopedic Society. The leader is Prof. Morio Matsumoto of Keio University. Nine representative hospitals in Japan that are famous for their scoliosis clinics joined it and collected DNAs of scoliosis patients, together with detailed clinical information necessary for genetic research. The consortium collected far more than 3,000 AIS subjects (4,109, as of January 12, 2016). With a help of this huge resource, we started the genetic study of AIS using a genome-wide association study (GWAS) method.

We conducted the GWAS using Illumina platforms for the Japanese population consisting of 1,050 AIS patients and 1,474 controls [2]. Inclusion criteria for case subjects were patients who saw expert scoliosis surgeons and: 1) the age at diagnosis was between 10–18 years, 2) had a Cobb angle of ≧ 15°, and 3) female. The control subjects were Japanese females. More than half million SNPs were successfully genotyped. After quality controls, data of 455,121 SNPs for 1,033 cases and 1,473 controls were analyzed for their association with AIS.

### 1^st^ locus

Three SNP on 10q24 showed genome-wide significance level in the GWAS [2]. The association of SNPs was replicated in independent Japanese female (326 cases and 9,823 controls) and male (94 cases and 1,849 controls) populations. The most significantly associated SNP was rs11190970. The *P*-value was 1.24 x 10^-19^ with odds ratio (OR) of 1.56 (95 % confidence interval (CI) = 1.41–1.71).

The association of rs11190970 was replicated in a Hong Kong Chinese population consisting of 300 cases and 788 controls (P = 9.10 x 10^-10^; OR (95 % CI) = 1.85 (1.52-2.25) [3]. The association was further replicated in Han Chinese in Nanjing [4] and Guangzhou [5]. We performed an international meta-analysis using Japanese, Han Chinese and Caucasian populations [6]. The meta-analysis using a total of 5,159 cases and 17,840 controls showed that rs11190870 is a global AIS susceptibility SNP (P = 1.22 x 10^-45^; OR = 1.60).

rs11190870 is in a 80 kb-LD block, which contains 2 genes (*LBX1*, *FLJ41350*). The two genes exist head to head on chromosome 10q, only 633 bp apart (database). rs11190870 is located 7.5 kb 3’ of *LBX1* (*lady bird late*, *Drosophila* homolog of, 1/ *lady bird*-like homeobox). *LBX1* is a homeobox gene, consisting of 2 exons and encodes a 280 amino-acid protein. It is specifically expressed during embryogenesis in mouse, with restricted expression to the developing central nervous system and muscles [7]. *Lbx1* knock-out (KO) mice had extensive muscle loss at birth [8, 9]. The KO mice also had defects in heart looping and myocardinal hyperplasia [10]. *Lbx1* is suggested to control the expression of genes that guide migrating muscle precursors and maintain their migratory potential. However, scoliosis was not found in the KO mice. On the other hand, neurons of the KO mice that arise in the dorsal spinal cord are eliminated by apoptosis [11]. Thus, *LBX1* could be implicated in myogenic and neurogenic etiology of AIS.

*FLJ41350* (alias: LOC399806 and *LBX1-AS1* (for *LBX1* antisense RNA 1)) is a hypothetical (predicted) gene. We cloned *FLJ41350* and determined its genomic structure. *FLJ41350* is predicted to encode a 120 amino-acid protein; its nucleotide and amino-acid sequences are human specific. Its predicted protein has no known homology to other human proteins.

To gain an insight into the role of *LBX1* and *FLJ41350* in the etiology of AIS, we examined their function *in vivo* using zebrafish. Human *LBX1* has three zebrafish homologues, namely, *lbx1a*, *lbx1b* and *lbx2* (Table 1)*.* Over-expression of the three *lbx* genes caused scoliosis, while *FLJ41350* did not (Guo *et al.* manuscript under review). Knockdown of the three *lbx* genes by morpholino (MO) also caused early onset scoliosis in zebrafish. Further studies are necessary to clarify the role of *LBX1* and *FLJ41350* in AIS etiology and pathogenesis.

**Table 1:** Amino-acid homology of human and zebrafish LBX genes

Gene	Amino acids	Homology (%)^a^ to	
		*LBX1*	*LBX2*
zebrafish			
*lbx1a*	269	72.2	39.4
*lbx1b*	265	59.9	38.9
*lbx2*	257	61.5	40.5
human			
*LBX1*	281	-	41.3
*LBX2*	198	41.3	-

^a^calculated by Needleman-Wunsch Global Sequence Alignment Tool (NCBI)

### 2^nd^ locus

To identify additional loci for AIS, we examined the association of top 30 SNPs in the GWAS. *P*-values of the SNPs were below 5.0 x 10^-5^. The replication study using 681 cases and 9,823 controls showed a significantly associated SNP, rs6570507 on chromosome 6q24.1 [12]. The combined *P*-value of the GWAS and the replication study was 6.96 × 10^-10^ (OR = 1.28). The association was replicated in Chinese and USA-Caucasian (non-Hispanic white) populations. The *P*-value in the meta-analysis was 1.27x 10^-14^ (OR = 1.27) [12].

**Fig. 1:**
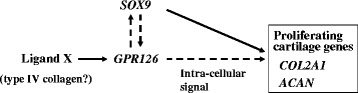
*GPR126*-related molecular network in cartilage. Solid and broken arrows indicate direct and indirect action

The most significantly associated SNPs were in intron 2 of *GPR126* (G protein-coupled receptor 126). *GPR126* consists of 26 exons and encodes a 1,250-aa orphan receptor. In humans, *GPR126* is highly expressed in cartilage. In mouse embryo, *Gpr126* is highly expressed in the proliferating cartilage of the spine, suggesting its role in spinal development [12]. *Gpr126* mRNA expression in early chondrogenic differentiation of ATDC5, a mouse model of chondrogenesis, showed that *Gpr126* increases with cartilage differentiation [13]. *Gpr126* over-expression increases expression of cartilage maker genes, *Col2a1* (encoding type II collagen) and *Acan* (encoding aggrecan), while *Gpr126* knock-down decreases their expression (Ikegawa *et al.* unpublished data). These data indicate that *Gpr126* is a positive regulator of cartilage differentiation (Fig. 1).

The association of *GPR126* SNPs with AIS is replicated in another Chinese population [14]. rs6570507 also showed association with trunk length in a meta-analysis of GWAS in European populations [15]. Conditional loss of *Gpr126* in chondrocyte lineages in mouse resulted in scoliosis and pectus excavatum [16]. Thus the scoliosis is post-natal onset and lacks malformations of vertebral units. The KO mouse would be a good model for AIS.

### 3^rd^ locus

The associations of the two loci were highly significant and were replicated in multiple association studies using different ethnic populations [2–6, 12]. The candidate genes in the loci are highly likely from their functional analysis [2, 12]. However, they only explain ~1 % of the total genetic variance in AIS; heritabilities (calculated as the square of the correlation coefficient, r^2^) of *LBX1* and *GPR126* were 1.0 % and 0.2 %, respectively.

To identify additional susceptibility gene(s) for AIS, we extended our GWAS by increasing the numbers of the subjects to more than 5 folds and conducting a whole genome imputation and a meta-analysis [17]. In total, we analyzed the association of 4,420,789 SNPs for 2,109 Japanese subjects with AIS and 11,140 control subjects. Three loci surpassed the genome-wide significance level of *P* < 5 × 10^−8^. Top two were the previously identified loci on 6q and 10q.

To confirm the association of the novel locus, we conducted a replication study with an independent Japanese cohort including 955 cases and 3,551 controls for 36 loci, yielding suggestive evidence of association (*P* < 1 × 10^−5^). In the replication study, we found evidence for association with one locus represented by a SNP, rs3904778 on chromosome 9p22.2. Combining the results of the GWAS and replication study, the *P* value of rs3904778 was 3.50 × 10^−11^ (OR = 1.21). We checked the association of rs3904778 in a Han Chinese population (1,268 cases and 1,173 controls). *P* value of the meta-analysis was 1.70 × 10^−13^ (OR = 1.21).

The most significantly associated SNPs were in intron 3 of *BNC2* that encodes a zinc finger transcription factor, basonuclin 2. eQTL data on public databases suggested that the associated SNPs have the potential to regulate *BNC2* transcription and the susceptibility alleles of the SNPs increase *BNC2* expression. By a series of electrophoretic mobility shift assay and reporter assay, we identified a functional SNP in *BCN2* (rs10738445), whose susceptibility allele had higher binding capacity to a transcription factor, YY1 (Yin yang 1) and higher *BNC2* enhancer activity than the non-susceptibility allele in the co-transfection experiment of the *BNC2* enhancer and *YY1. Bnc2* over-expression in zebrafish embryo produced axial body curvature associated with abnormal somite formation. The *in vitro* and *in vivo* evidence strongly suggest that increased *BNC2* expression predisposes to AIS.

### Association with severity

The factors that influence the progression of scoliosis are clinically very important, as the AIS treatment depends on severity and progression. These factors are also reported to have genetic components [18, 19]: the meta-analysis of a Danish twin study reported that a significant correlation with curve severity was found in monozygous twins, but not in dizygous twins [20]. SNPs in *ESR1*, *ESR2*, *MATN1*, and *IGF1* genes are reported to be associated with AIS severity [21–24].

Therefore, we performed a GWAS by using only severely affected AIS subjects (Cobb’s angle above 40°). Through a two-stage association study using a total of ~12,000 Japanese subjects, we identified six SNPs of genome-wide significance level association. Five of them were in the known loci of AIS susceptibility that we previously reported: three were close to *LBX1* on chromosome 10q24.31 [2] and two on chromosome 6q24.1 in *GPR126* [12]. We examined the correlation between risk allele frequency of SNPs on the *LBX1* and *GPR126* loci, but found that both loci had no correlation with AIS severity (Table 2). Therefore, we considered that these SNPs determine initiation (susceptibility), but not progression/severity.

**Table 2:** Severity of AIS and risk allele frequency of previously identified associated SNPs

Group		rs11190870 (*LBX1*)		rs6570507 (*GPR126*)	
		No. subject	RAF (%)	No. subject	RAF (%)
Patient	*Cobb angle*				
	>50	407	67.1	491	50.5
	40 – 50	278	66.7	352	49.1
	30 – 40	270	66.9	440	46.6
	20 – 30	363	67.1	458	49.1
	15 – 20	41	67.1	7	45.5
General control		11,294	56.5	25,939	42.9

RAF, risk allele frequency

The remaining one, rs12946942 is on chromosome 17q24.3, and showed a significant association in the recessive model (*P* = 4.00 × 10^-8^, OR = 2.05). The association of the SNP was replicated in a Han Chinese population (meta-analysis combined *P* = 6.43 × 10^-12^, OR = 2.21). rs12946942 and the LD block containing it are in the gene desert region. The closest genes are *SOX9* (MIM 608160) and *KCNJ2* (MIM 600681). Both of them are ~1 Mb away from the SNP, but are excellent candidate genes.

*SOX9* is the master transcription factor of cartilage [25]. *SOX9* mutations cause campomelic dysplasia (MIM 114290), a skeletal dysplasia characterized by bowing of the long bones, small scapula, tracheobronchial narrowing, sex reversal, and kyphoscoliosis [26]. Very long-range cis-regulatory elements controlling tissue-specific *SOX9* expression have been reported [27, 28]. The LD block containing rs12946942 has recently been defined as a susceptibility locus of prostate cancer [29]. The block contains six enhancer elements, of which the E1 enhancer forms a long-range chromatin loop to *SOX9* in a prostate cancer cell line. Two SNPs within the E1 enhancer were shown to direct allele-specific gene expression. rs12946942 may likewise participate in scoliosis pathogenesis by controlling scoliosis-related tissue-specific *SOX9* expression.

*KCNJ2* encodes a potassium channel, a component of the inward rectifier current IK1 [30]. *KCNJ2* mutations cause a cardiodysrhythmic type of periodic paralysis known as Andersen-Tawil syndrome (ATS; MIM 170390) [31]. The syndrome is characterized by ventricular arrhythmias, periodic paralysis, facial and skeletal dysmorphism including hypertelorism, small mandible, cleft palate, syndactyly, clinodactyly, and scoliosis [30, 31]. Furthermore, the 17q24.2-q24.3 micro-deletion syndrome whose deletion includes *KCNJ2* and rs12946942 exhibited skeletal malformations similar to ATS, including progressive scoliosis [32]. However, a similar micro-deletion that includes *KCNJ2*, but not rs12946942, has no scoliosis phenotype [33]. Further studies are necessary to identify the causal gene in the locus.

## Conclusions

### International collaboration

I believe we need to identify more genes to clarify the whole picture of AIS. The total genetic variance in AIS explained by the three genes is still < 2 %. By increasing the number of samples, we can expect to increase the power of the association study. As in the other fields of GWASs, international collaboration for AIS genetic study is now in progress. An international consortium, ICSG (International Consortium for Scoliosis Genetics) has been established since 2012. Prof. Carol Wise (Texas Scottish Rite Hospital for Children) organized the consortium. Large-scale trans-ethnic association studies including GWASs will facilitate the identification of AIS susceptibility genes. We can see the good example of such collaboration in the GWAS of rheumatoid arthritis [34].

#### Association to function

Association does not mean causality. GWAS just shows a marker on genome, not a disease-causing sequence variation (causal variant). After all, a result of an association study is just a statistic. We must find a causal variant. We must clarify pathogenesis of AIS through functional studies. We must convert statistics to biology, and biology to medicine. Otherwise, we cannot reach to our ultimate goal, the treatment of AIS. Therefore, just like scoliosis, a long and winding road toward the truth of AIS is still before us.
